# The word-with-noise test: test-retest reliability in normal-hearing adults

**DOI:** 10.1590/2317-1782/20232023093en

**Published:** 2024-04-08

**Authors:** Lidiéli Dalla Costa, Ana Valéria de Almeida Vaucher, Maristela Julio Costa

**Affiliations:** 1 Universidade Federal de Santa Maria - UFSM - Santa Maria (RS), Brasil.

**Keywords:** Hearing, Speech Perception, Hearing Tests, Noise, Reproducibility of Results, Psychometrics, Adult

## Abstract

**Purpose:**

To investigate the reliability of the Word-with-Noise Test in a group of normal-hearing adults.

**Methods:**

Forty-five normal-hearing adult subjects participated in the research. The interval between the first and second assessment was 14 to 28 days, performed during the same time of the day and by the same evaluator. The comparison analysis between the test and the retest was performed considering the general result of the ears, totaling 90 ears evaluated. The inferential analysis included the comparison of the situations in the first and second assessment using the Wilcoxon Test, calculation, and interpretation of the Intraclass Correlation Index.

**Results:**

There was a statistically significant difference between the test and retest performances. The intraclass correlation coefficients obtained were indicative of good reliability (r=0.759; p<0.001) for the monosyllabic stimulus and moderate reliability (r=0.631; p<0.001) for the disyllabic stimulus.

**Conclusion:**

The Word-with-Noise Test demonstrated satisfactory reliability for both the monosyllabic and disyllabic stimuli.

## INTRODUCTION

One of the main auditory complaints reported by subjects treated in audiology services is the difficulty in recognizing speech in the presence of competitive noise^(1)^. Speech recognition is commonly assessed through speech perception assessment, which is performed in a quiet environment. However, although the results obtained through speech perception assessment are essential for audiological diagnosis, several audiologist researchers defend the idea that these results do not allow measuring the subject’s hearing difficulty in everyday communicative situations, which generally occur in noisy environments^(2-5)^.

The word recognition task in noise adds a significant cognitive load compared to a similar task in a quiet environment. Speech recognition tests in noise can be considered a “stress assessment” of the auditory function^(6)^.

As a result, numerous studies highlight the importance of using speech-in-noise tests in clinical practice with the argument that hearing difficulty should be assessed in addition to pure tone audiometry and speech audiometry in a quiet environment, which needs to be complemented by testing the speech perception in noise^(2-7)^. The use of speech-in-noise tests is also suggested by professional guidelines that consider this type of test an essential tool in audiological assessment^(8,9)^, which helps in organizing professional conduct and counseling patients with this type of complaint.

A possible test to be used to complement the basic audiological battery is the Words-with-Noise (WWN) Test^(10)^, composed of two lists of monosyllabic words (WWN-M)^(11-13)^, five lists of disyllabic words (WWN-D)^(14,15)^, with each list consisting of 25 words and speech spectrum noise^(16)^, developed according to pre-established criteria and with defined psychometric measures^(17)^.

Taking into account that the entire assessment instrument has to be considered valid and reliable in order to allow more precise decisions and increase scientific rigor in the interpretation of its results^(18)^, the WWN Test was developed considering the validity requirements, but still requires reliability evidence. Reliability is one of the main quality criteria of an instrument and reflects its ability to reproduce a result consistently over time^(19)^. This parameter can be assessed through test-retest, that is, the degree to which similar results are obtained at two different times in the same population^(20)^.

In order to continue the psychometric studies on this new instrument, this research aimed to seek evidence of the WWN Test reliability.

## METHODS

This is a prospective, descriptive study carried out at a Higher Education Institution and approved by the institution's Ethics and Research Committee, under no. 3.660.209. All research participants signed the Free Consent Form (FCF).

The sample for this study was taken by convenience. Participants were recruited through an invitation published on social networks and a verbal invitation from the researcher.

The inclusion criteria for the sample composition were: being between 19 and 44 years old; air conduction thresholds lower than 20 dBHL at frequencies from 250 to 8000 Hz; having at least completed primary education, and being right-handed. The exclusion criteria were: presenting hearing complaints; middle ear changes; evident health changes that could compromise the procedures (neurological, psychological, mental or cognitive disorders), and/or noticeable speech changes.

Initially, 50 individuals were recruited and underwent the first assessment. Of these, 45 subjects returned for the second assessment, thus resulting in a loss of 10% of the sample. Thus, the sample group consisted of 11 men (24.4%) and 34 women (75.6%), with an average age of 25.91 years.

### Instruments and Procedures

In the first assessment, an anamnesis was carried out to investigate personal data, education level, otological history, and hearing complaints. The reduced version of the Edinburgh Handedness Inventory, modified^(21)^ and validated for the Brazilian population^(22)^, was also applied through interviews in order to confirm each subject’s handedness.

Subsequently, a visual inspection of the external auditory canal of both ears was carried out and, once any alteration that could interfere with the expected assessments had been ruled out, the participants underwent evaluation of acoustic immittance measurements, pure tone audiometry and, finally, were evaluated with monosyllabic and disyllabic WWN Test.

Acoustic immittance measurements were carried out using the Interacoustics AT 235 tympanometer. The pure-tone audiometry and the WWN Test application were performed using the Interacoustics AC 33 audiometer, and TDH 39 earphones, in an acoustically treated environment. In addition, one also used a Toshiba CD-4149 Player coupled to the audiometer to present speech and noise stimuli in digital recording. To obtain measurements with the WWN Test, the word lists and competitive noise were applied by using earphones, with the different stimuli being presented monaurally.

To ensure the accuracy of the measurements, before the test was applied, the equipment was calibrated for each subject separately for each channel based on the audiometer VU-meter, which was adjusted to zero. Thus, a pure tone of 1 kHz was used to calibrate the channel on which the words were recorded, while to calibrate the noise channel, as it is a continuous stimulus, the very noise used in the research was used.

All monosyllabic and disyllabic lists were applied randomly. The order of presentation of the lists according to the side of the ear was carried out alternately, following the classification of subjects evaluated as *odd* and *even*, starting with the assessment of the right ear in even subjects and the left ear in odd subjects.

Considering this, the WWN Test word lists were presented in the following order:

1. Initially, the calibration of the different channels of the test equipment was carried out;
2. Presentation of the application strategy and requested response to perform the WWN Test in the ear chosen to start with;
3. To familiarize the subject with the test, the first 10 monosyllabic words from the training list were applied in the presence of noise in the opposite ear;
4. Alternating the side of the ear again, a list of monosyllables was presented in the presence of competitive noise;
5. Next, a list of disyllables was presented to the same ear, also in the presence of noise;
6. Alternating the side of the ear once more, a list of monosyllables was presented in the presence of noise;
7. Finally, on the same side of the ear, a list of disyllables was presented in the presence of noise.

The WWN Test application strategy was defined as researching the Speech Recognition Threshold (SRT) with noise fixed at 55 dBHL. To obtain the SRT, the sequential or adaptive or ascending-descending technique was used^(23)^, which allows obtaining the level of stimulus presentation, in which a subject can recognize approximately 50% of the speech stimuli in a given condition^(24)^.

To obtain the SRTs in noise, the 10 words from the training list were initially presented at an S/N ratio of + 10 dBHL. Next, the test began with the presentation of the first word of each list applying 10 dBHL above the level at which the first incorrect response in the training list occurred, seeking to ensure that the subject was able to recognize correctly the first word in the list. This strategy seeks to minimize the variability of responses and also motivate the subject under evaluation.

Continuing, the presentation level was successively reduced by 4 dBHL and the next words were presented until the response was reversed, that is, when the subject’s response was incorrect. From that point on, the presentation levels changed to intervals of 2 dBHL, which were increased when there was an incorrect response and decreased when there was a correct response.

The subjects were instructed to repeat each word immediately after hearing it, and in cases in which the individual presented two similar words as a response due to not being sure about it, the first repeated word was considered.

For data analysis, the presentation levels of each word in the lists were recorded and, then, to calculate the SRT in the presence of noise obtained for each list, the mean was calculated based on the presentation level at which the first incorrect response occurred up to the presentation level of the last word in the list. Finally, to calculate the S/N ratio, the calculated SRT was subtracted from the noise presentation level, which in this study was 55 dBHL.

The retest was administered 14 to 28 days after the first assessment, on the same period of the day and by the same evaluator. In this second assessment, a brief anamnesis was administered, seeking to verify the occurrence of any situation during this period between assessments that could influence the subject’s performance in the reassessment related to hearing or any significant emotional aspect. A new visual inspection of the external auditory canal and acoustic immittance measurements were also carried out, in order to rule out any changes in the external and middle ear that could have been acquired in the period between assessments. Subsequently, the subjects were Assessed with the WWN Test as performed in the first assessment.

### Data analysis

The information collected was tabulated and then analyzed and compared in a descriptive and inferential statistical manner, in accordance with the proposed objectives. Statistical analyzes were carried out using SPSS V20, Minitab 16 and Excel Office 2010. Non-parametric statistical tests were used, as the study variables, which were analyzed using the Kolmogorov-Smirnov test (N≥30), presented a non-normal distribution. A significant result was considered p ≤ 0.05, with a 95% confidence interval.

The inferential analysis included a comparison of test and retest situations using the Wilcoxon Test, calculation and interpretation of the Intraclass Correlation Index. Intraclass Correlation Indexes below 0.5, between 0.5 and 0.75, and between 0.75 and 0.9 were seen as indicative of weak, moderate, good, or excellent reliability, respectively^(25)^.

## RESULTS

Initially, a comparison was made between the ears evaluated considering the side of the ear (right and left). No statistically significant differences were observed in the ear side variable for monosyllabic words in the test (p=0.321) and retest (p=0.949) conditions, and disyllabic words in the test (p=0.182) and retest (p=0.937) conditions. Thus, this verification allowed the comparison analysis between the test and retest to be carried out considering the general result of the ears, totaling 90 ears evaluated.

When comparing performance in the test and retest, a statistically significant difference was observed between the first and second assessment, and, when observing the average S/N ratios, values were verified in more unfavorable conditions in the retest, both for monosyllables and for disyllables. Nevertheless, the differences between the average values obtained in the test and retest were less than 1 dB (Table 1).

**Table 1:** Descriptive values and comparative analysis of the Words-with-Noise Test in test and retest situations

		**N**	**Mean**	**Median**	**Standard deviation**	**Q1**	**Q3**	**CI**	**P-value**
Monosyllables	Test	90	-2.35	-2.39	1.89	-3.80	-1.04	0.39	<0.001*
	Re-test	90	-3.14	-3.13	1.81	-4.49	-2.00	0.37	
Disyllables	Test	90	-5.95	-6.13	1.64	-7.19	-4.67	0.34	0.029*
	Re-test	90	-6.38	-6.39	1.81	-7.60	-5.17	0.37	

Wilcoxon test

* Statistically significant value at the 5% level (p ≤ 0.05)

Results expressed in Signal/Noise ratio

**Caption:** N: number of ears; Q: quartile; CI: confidence interval

**Figure 1:**
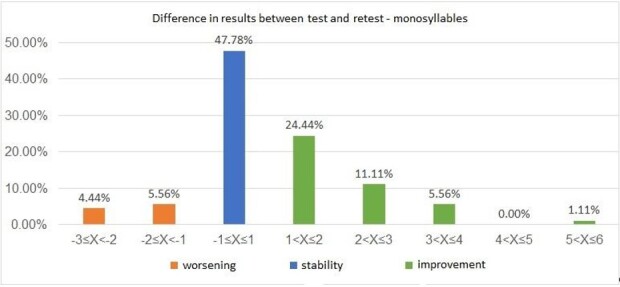
Distribution of the difference in results between the test and retest in the Words-with-Noise Test with monosyllabic stimuli (n=90)

**Figure 2:**
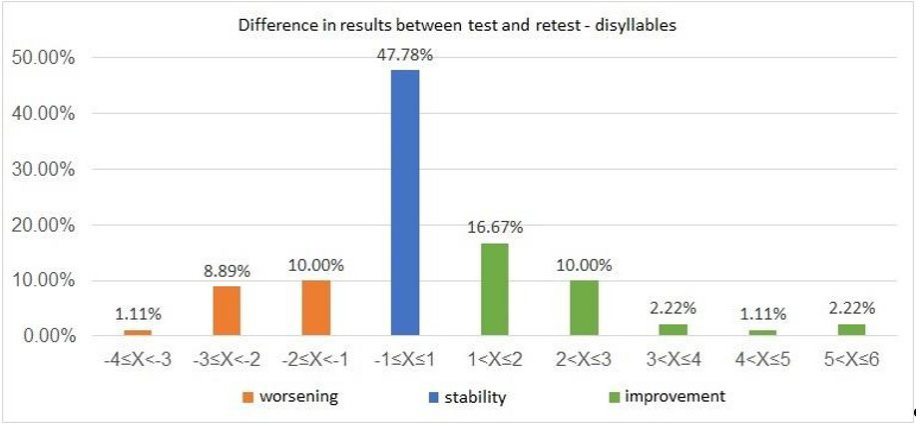
Distribution of the difference in results between the test and retest in the Words-with-Noise Test with disyllabic stimulus (n=90)

An analysis of the differences between the results obtained in the first and second assessment (retest result - test result) was also carried out, both for the monosyllabic stimulus (Figure 1) and for the disyllabic stimulus (Figure 2). It was possible to observe that 90% (n= 81) of the results showed stability or improved performance in the retest in the assessment carried out with monosyllables, and 80% (n= 72) also demonstrated this behavior with disyllables.

**Caption:** X: difference between test and retest results, expressed in dB;.

Worsening: difference between test and retest results less than -1 dB;

Stability: difference between test and retest results between -1 dB and 1 dB;

Improvement: difference between test and retest results greater than 1 dB

**Caption:** X: difference between test and retest results, expressed in dB;

Worsening: difference between test and retest results less than -1 dB;

Stability: difference between test and retest results between -1 dB and 1 dB;

Improvement: difference between test and retest results greater than 1 dB

Regarding the reliability analysis in the test-retest for the WWN Test (Table 2), the results demonstrated a good degree of reliability for monosyllables and moderate for disyllables, which are considered statistically significant.

**Table 2:** Analysis of test-Retest reliability of the Words-with-Noise Test

	ICI	Inferior Lim.	Superior Lim.	P-value
Monosyllables	0.759	0.548	0.861	<0.001*
Disyllables	0.631	0.441	0.757	<0.001*

Intraclass Correlation Index(ICI)

* Statistically significant value at the 5% level (p ≤ 0.05)

**Caption:** Lim: limit

## DISCUSSION

With the purpose of assessing the WWN Test reliability, an analysis of the normal-hearing subjects’ performance in the test-retest was carried out. Such analysis is considered one of the main quality criteria of an instrument, and one of the most used strategies in the health area to verify reliability^(18)^.

When analyzing the WWN Test results, it was possible to verify that, despite the significant difference observed between the test and retest (Table 1), there was a tendency towards stability in the measurements obtained at different moments of the assessment or, alternatively, towards an improvement in the results for both stimuli, with data concentration occurring between -1 to +3 dB (Figures 1 and 2).

The test-retest reliability analysis for the WWN Test (Table 2) was carried out using the Intraclass Correlation Index, which is one of the main statistical tests used to estimate the stability of the results of an instrument, as it takes into account the measurement errors^(26)^. The results demonstrated a good degree of agreement between the subjects’ performance in the test and retest for monosyllables and a moderate degree of agreement for disyllables.

A study analyzed the test and retest reliability of an instrument that uses monosyllabic words to evaluate speech recognition in noise - Speech in Babble (SiB) test - in normal-hearing adults, by applying the test twice to the same ear in a single assessment moment^(27)^, and no statistically significant difference was found between the two tests, both in the right and in the left ears. The researchers stated that test-retest reliability was “equivalent”.

The reliability of an instrument assessed through test-retest is not considered a fixed property, given that there are some sources of error that are beyond the evaluator’s control. In retest assessments, the ideal is that subjects maintain the performance of the first assessment^(28)^; however, speech recognition assessments are extremely sensitive to each person’s intrinsic factors. Improvements in the second assessment may be associated with familiarization with the test (application and stimulus strategy), as well as with the possibility of learning^(20)^. Worse performance on the retest may be associated with the level of attention and concentration, lack of motivation due to the fact that the test is no longer new, and stress, among other intrinsic factors^(20)^.

Another issue to be considered is the fact that the WWN Test is composed of monosyllabic and disyllabic words, which are sensitive to each subject’s auditory abilities. The word stimulus represents small units of speech, which have a certain linguistic context, are familiar, but have few acoustic clues and little redundancy, which means that subjects need to hear most of their elements to recognize them^(29)^. Therefore, words are more susceptible to errors due to intrinsic factors, such as attention and concentration issues, as the “loss” of acoustic information from a single phoneme already makes word recognition difficult.

Methodological factors can also influence test-retest results, such as the interval between the first and second assessment, and sample size. Seeking to reduce the influence of these factors, this study took into account the main methodological recommendations for researching the instrument reliability^(18,20,28)^.

Regarding the interval between testing and retesting, it is known that the period between test repetition should be long enough to prevent the memory effect, but short enough to ensure that clinical changes do not occur, influencing its interpretation^(18,20)^. Therefore, a time interval of 14 to 28 days was chosen, considered appropriate for this purpose.

Regarding the sample size, this study had a sample of 45 assessed subjects, close to the recommendation of researchers who suggest samples greater than 50 participants to assess the test-retest reliability^(28)^ and exceeding the minimum recommendation of 20 subjects for analysis of the Intraclass Correlation Coefficient^(30)^.

Despite the variability observed in the second assessment, the correlation analysis revealed a good degree of agreement between test and retest for WWN-M, and a moderate degree of agreement for WWN-D. This indicates acceptable reliability of the results obtained with the WWN Test, considering the inherent variability of speech recognition assessment instruments and also the main objective of applying the WWN Test in clinical routine, which is to be an instrument capable of identifying the difficulty for a subject to recognize speech in noise and, based on its results, verify if additional assessments are necessary.

However, if the examiner aims to use it in a situation of comparison before and after auditory training or adaptation of sound amplification, one should take into account that a subject, when reevaluated with a test with such characteristics, may present variability in performance in the second assessment. Therefore, its results should be analyzed with caution.

Regarding the limitations of the study, it is possible to list the difficulty controlling each subject’s intrinsic conditions, such as emotional state, attention and concentration, which can interfere with the results of a behavioral test when it has to be applied at different moments of assessment.

## CONCLUSION

Words-with-Noise (WWN) Test demonstrated a satisfactory degree of reliability in the test-retest in normal-hearing adults, both for the monosyllabic stimulus and for the disyllabic stimulus.
