# Supplementary material for: The word-with-noise test: test-retest reliability in normal-hearing adults
Source: Codas. 2024 Apr 8;36(2):e20230093. doi: 10.1590/2317-1782/20232023093en (PMC11042685; doi:10.1590/2317-1782/20232023093en)
Supplement: Translation

# Translation: Teste de palavras no ruído: confiabilidade no teste-reteste em adultos normo-ouvintes

**DOI:** 10.1590/2317-1782/20232023093pt

**Authors:** Lidiéli Dalla Costa, Ana Valéria de Almeida Vaucher, Maristela Julio Costa

**Affiliations:** 1 Universidade Federal de Santa Maria - UFSM - Santa Maria (RS), Brasil.

**Keywords:** Audição, Percepção da Fala, Testes Auditivos, Ruído, Reprodutibilidade dos Testes, Psicometria, Adulto

## Abstract

**Objetivo:**

Investigar a confiabilidade do Teste de Palavras no Ruído em um grupo de adultos normo-ouvintes.

**Método:**

Participaram da pesquisa 45 sujeitos adultos normo-ouvintes. O intervalo entre a primeira e a segunda avaliação foi de 14 a 28 dias, realizadas no mesmo turno do dia e pelo mesmo avaliador. A análise de comparação entre teste e reteste foi realizada considerando o resultado geral das orelhas, totalizando 90 orelhas avaliadas. A análise inferencial incluiu a comparação das situações na primeira e segunda avaliação por meio do Teste de Wilcoxon, cálculo e interpretação do Índice de Correlação Intraclasse.

**Resultados:**

Houve diferença estatisticamente significante entre os desempenhos no teste e reteste. Os coeficientes de correlação intraclasse obtidos foram indicativos de boa confiabilidade (r=0,759; p<0,001) para o estímulo monossilábico e de moderada confiabilidade (r=0,631; p<0,001) para o dissilábico.

**Conclusão:**

O Teste de Palavras no Ruído demonstrou satisfatória confiabilidade tanto para o estímulo monossilábico, quanto para o dissilábico.

## INTRODUÇÃO

Uma das principais queixas auditivas referidas pelos sujeitos atendidos em serviços de audiologia é a dificuldade de reconhecer a fala na presença de ruído competitivo^(^1^)^. O reconhecimento de fala é comumente avaliado por meio da logoaudiometria, a qual é realizada em ambiente silencioso. Porém, apesar dos resultados obtidos por meio da logoaudiometria serem essenciais para o diagnóstico audiológico, diversos pesquisadores audiologistas defendem a ideia de que esses resultados não permitem dimensionar a dificuldade auditiva do sujeito nas situações comunicativas cotidianas, as quais geralmente ocorrem em ambientes ruidosos^(^2-5^)^.

A tarefa de reconhecimento de palavras no ruído adiciona uma carga cognitiva significativa em comparação com uma tarefa semelhante em ambiente silencioso. Testes de reconhecimento de fala no ruído podem ser considerados como uma “avaliação de estresse” da função auditiva^(^6^)^.

Em função disso, numerosos estudos ressaltam a importância da utilização dos testes de fala no ruído na prática clínica com o argumento de que a dificuldade auditiva deve ser avaliada além da audiometria de tons puros e de fala em ambiente silencioso, que precisa ser complementada testando a percepção de fala no ruído^(^2-7^)^. A utilização de testes de fala no ruído também é sugerida por diretrizes profissionais que consideram esse tipo de teste uma ferramenta essencial na avaliação audiológica^(^8,9^)^, a qual auxilia na organização da conduta profissional e no aconselhamento do paciente com este tipo de queixa.

Uma possibilidade de teste para ser utilizado na complementação da bateria audiológica básica é o Teste de Palavras no Ruído - TPR^(^10^)^ composto por duas listas de vocábulos monossilábicos (TPR-M)^(^11-13^)^, cinco listas de vocábulos dissilábicos (TPR-D)^(^14,15^)^, sendo cada lista composta por 25 vocábulos e um ruído com espectro de fala^(^16^)^, desenvolvido atendendo a critérios preestabelecidos e com medidas psicométricas definidas^(^17^)^.

Levando em consideração que todo o instrumento de avaliação deve ser considerado válido e confiável, a fim de permitir decisões mais precisas e aumentar o rigor científico na interpretação de seus resultados^(^18^)^, o TPR foi desenvolvido considerando os requisitos de validade, porém ainda requer evidências de confiabilidade. A confiabilidade é um dos principais critérios de qualidade de um instrumento e reflete a sua capacidade de reproduzir um resultado de forma consistente no tempo^(^19^)^. Esse parâmetro pode ser avaliado por meio do teste-reteste, ou seja, o grau em que resultados similares são obtidos em dois momentos distintos na mesma população^(^20^)^.

A fim de dar continuidade aos estudos psicométricos desse novo instrumento, a presente pesquisa teve como objetivo buscar evidências de fidedignidade do TPR.

## MÉTODO

Trata-se de um estudo de caráter prospectivo descritivo realizado em uma Instituição de Ensino Superior e aprovado pelo Comitê de Ética e Pesquisa da instituição, sob o número 3.660.209. Todos os participantes do estudo assinaram o Termo de Consentimento Livre e Esclarecido (TCLE).

A amostra deste estudo ocorreu por conveniência. O recrutamento dos participantes aconteceu por meio de divulgação de convite em redes sociais e contato pessoal do pesquisador.

Os critérios de inclusão para a composição da amostra foram: apresentar idade entre 19 e 44 anos; limiares auditivos de via aérea inferiores a 20 dBNA nas frequências de 250 a 8000 Hz; possuir no mínimo o ensino fundamental completo e apresentar preferência manual direita. Os critérios de exclusão foram: apresentar queixas auditivas; alteração de orelha média; alterações de saúde evidentes que pudessem comprometer a execução dos procedimentos (distúrbios neurológicos, psicológicos, mentais ou cognitivos) e/ou alterações de fala perceptíveis.

Inicialmente, foram avaliados 50 indivíduos, os quais foram submetidos a primeira avaliação. Desses, 45 sujeitos retornaram para a segunda avaliação, ocorrendo, assim, perda de 10% da amostra. Dessa forma, o grupo amostral ficou constituído por 11 indivíduos do sexo masculino (24,4%) e 34 do sexo feminino (75,6%), com média de idade de 25,91 anos.

### Instrumentos e procedimentos

Na primeira avaliação, foi aplicada uma anamnese direcionada para investigação dos dados pessoais, nível de escolaridade, história otológica e queixas auditivas. Também foi aplicado por meio de entrevista a versão reduzida do Teste de Dominância Manual de Edinburgh, modificado^(^21^)^ e validado para população brasileira^(^22^)^, a fim de confirmar a dominância manual de cada sujeito.

Posteriormente, foi realizada a inspeção visual do meato acústico externo de ambas as orelhas e, uma vez descartada qualquer alteração que pudesse interferir nas avaliações previstas, os participantes foram submetidos à avaliação das medidas de imitância acústica, audiometria tonal liminar e, finalmente, foram avaliados com o TPR monossilábico e dissilábico.

As medidas de imitância acústica foram realizadas utilizando-se o imitanciômetro da marca *Interacoustics* modelo AT 235. Já a realização da audiometria tonal liminar e a aplicação do TPR foram executadas utilizando-se o audiômetro da marca *Interacoustics,* modelo AC 33, e fones auriculares, modelo TDH 39, em ambiente tratado acusticamente. Além disso, um *Compact Disc Player*, da marca Toshiba, modelo CD-4149, acoplado ao audiômetro para apresentação dos estímulos de fala e do ruído em gravação digital. Para a obtenção das medidas com o TPR, as listas de palavras e o ruído competitivo foram aplicados por meio de fones auriculares, sendo que os diferentes estímulos foram apresentados de forma monoaural.

Para garantir a exatidão das medidas, antes de iniciar a aplicação do teste, para cada sujeito foi realizada a calibração do equipamento para cada canal separadamente com base no *VU-meter*do audiômetro, que foi ajustado no nível zero. Assim, para calibrar o canal no qual estavam gravadas as palavras foi usado um tom puro de 1 kHz, enquanto que para calibrar o canal do ruído, por ser um estímulo contínuo, foi empregue o próprio ruído utilizado na pesquisa.

Foram aplicadas todas as listas mono e dissilábicas de forma randomizada. A ordem de apresentação das listas segundo o lado da orelha foi realizada de forma alternada, seguindo a classificação dos sujeitos avaliados em pares e ímpares, iniciando pela avaliação da orelha direita nos sujeitos pares e pela orelha esquerda nos sujeitos ímpares.

Considerando isso, as listas de palavras do TPR foram apresentadas de acordo com a seguinte ordem:

1. Inicialmente, foi realizada a calibração dos diferentes canais do equipamento de teste;
2. Apresentação da estratégia de aplicação e resposta solicitada para a realização do TPR na orelha escolhida para iniciar o teste;
3. Para familiarização do sujeito com o teste, foram aplicadas as 10 primeiras palavras monossilábicas da lista treino na presença do ruído na orelha oposta;
4. Alternando novamente o lado da orelha, foi apresentada uma lista de monossílabos na presença de ruído competitivo;
5. A seguir, na mesma orelha foi apresentada uma lista de dissílabos também na presença de ruído;
6. Alternando novamente o lado da orelha, foi apresentada uma lista de monossílabos na presença de ruído;
7. Finalmente, no mesmo lado da orelha foi apresentada uma lista de dissílabos na presença de ruído.

A estratégia de aplicação do TPR consiste na pesquisa do Limiar de Reconhecimento de Fala (LRF) com ruído fixo em 55 dBNA. Para a obtenção do LRF foi utilizada a técnica sequencial ou adaptativa ou ainda ascendente-descendente^(^23^)^, a qual permite obter o nível de apresentação do estímulo, no qual um sujeito pode reconhecer aproximadamente 50% dos estímulos de fala em uma determinada condição^(^24^)^.

Para a obtenção dos LRFs no ruído, inicialmente foram apresentadas as 10 palavras da lista treino em uma relação S/R de + 10 dBNA. A seguir, iniciou-se a aplicação do teste com a apresentação da primeira palavra de cada lista 10 dBNA acima do nível no qual ocorreu a primeira resposta incorreta na lista treino, buscando garantir que o sujeito fosse capaz de reconhecer corretamente a primeira palavra da lista. Essa estratégia busca minimizar a variabilidade das respostas e também motivar o sujeito avaliado.

Dando continuidade, foi diminuído sucessivamente o nível de apresentação em 4 dBNA e apresentadas as próximas palavras até haver reversão da resposta, ou seja, quando o sujeito respondeu de forma incorreta. Desse ponto em diante, os níveis de apresentação passaram a intervalos de 2 dBNA, que foram aumentados quando houve erro na resposta e diminuídos quando houve acerto.

Os sujeitos foram instruídos a repetir cada palavra imediatamente após ouvi-la, sendo que nos casos nos quais o indivíduo apresentou como resposta duas palavras semelhantes, por haver incerteza na resposta, foi considerada a primeira palavra repetida.

Para a análise dos dados, foram registrados os níveis de apresentação de cada palavra das listas e, então, para calcular o LRF na presença de ruído obtido para cada lista foi calculada a média a partir do nível de apresentação no qual ocorreu a primeira resposta incorreta até o nível de apresentação da última palavra da lista. Finalmente, para o cálculo da relação S/R o LRF calculado foi subtraído do nível de apresentação do ruído, que neste estudo foi de 55 dBNA.

O reteste foi aplicado em um intervalo de 14 a 28 dias após a primeira avaliação, no mesmo turno do dia e pelo mesmo avaliador. Nessa segunda avaliação, foi aplicada uma breve anamnese que buscou questionar sobre a ocorrência de alguma situação nesse período entre as avaliações que pudesse influenciar no desempenho do sujeito na reavaliação relacionada à audição ou algum aspecto emocional significativo. Também foi realizada uma nova inspeção visual do meato acústico externo e medidas de imitância acústica, a fim de descartar qualquer alteração de orelha externa e média que pudesse ter sido adquirida no período entre as avaliações. Posteriormente, os sujeitos foram avaliados com o TPR conforme executado na primeira avaliação.

### Análise dos dados

As informações coletadas foram tabuladas e, então, analisadas e comparadas de maneira descritiva e estatística inferencial, de acordo com os objetivos propostos. Para as análises estatísticas foram utilizados os programas SPSS V20, Minitab 16 e Excel Office 2010. Foram utilizados testes estatísticos não paramétricos, pois as variáveis do estudo, as quais foram analisadas por meio do teste de Kolmogorov-Smirnov (N≥30), apresentaram distribuição não normal. Foi considerado resultado significante p ≤ 0,05, com confiança de 95%.

A análise inferencial incluiu comparação das situações de teste e reteste por meio do Teste de Wilcoxon, cálculo e interpretação do Índice de Correlação Intraclasse. Índices de Correlação Intraclasse abaixo de 0,5, entre 0,5 e 0,75, entre 0,75 e 0,9 foram considerados, respectivamente, indicativos de confiabilidade fraca, moderada, boa ou excelente^(^25^)^.

## RESULTADOS

Inicialmente, foi realizada a comparação entre as orelhas avaliadas considerando o lado da orelha (direita e esquerda). Não foram observadas diferenças estatisticamente significantes na variável lado da orelha para os vocábulos monossilábicos na condição teste (p=0,321) e reteste (p=0,949), e dissilábicos na condição teste (p=0,182) e reteste (p=0,937). Assim, tal verificação possibilitou que a análise de comparação entre teste e reteste fosse realizada considerando o resultado geral das orelhas, totalizando 90 orelhas avaliadas.

Ao comparar o desempenho no teste e reteste, observou-se diferença estatisticamente significante entre a primeira e segunda avaliação, sendo que, ao observar as relações S/R médias, foram verificados valores em condições mais desfavoráveis no reteste, tanto para os monossílabos quanto para os dissílabos. Porém, as diferenças entre os valores médios obtidos no teste e reteste foram menores do que 1 dB (Tabela 1).

**Tabela 1:** Valores descritivos e análise comparativa do Teste de Palavras no Ruído nas situações teste e reteste

		**N**	**Média**	**Mediana**	**Desvio Padrão**	**Q1**	**Q3**	**IC**	**p-valor**
Monossílabos	Teste	90	-2,35	-2,39	1,89	-3,80	-1,04	0,39	<0,001*
	Reteste	90	-3,14	-3,13	1,81	-4,49	-2,00	0,37	
Dissílabos	Teste	90	-5,95	-6,13	1,64	-7,19	-4,67	0,34	0,029*
	Reteste	90	-6,38	-6,39	1,81	-7,60	-5,17	0,37	

Teste Wilcoxon

* Valor estatisticamente significante no nível de 5% (p ≤ 0,05)

Resultados expressos em relação Sinal/Ruído

**Legenda:** N: número de orelhas; Q: quartil; IC: Intervalo de confiança

Também foi realizada a análise das diferenças entre os resultados obtidos na primeira e segunda avaliação (resultado reteste - resultado teste), tanto para o estímulo monossilábico (Figura 1) quanto para o dissilábico (Figura 2). Foi possível observar que 90% (n= 81) dos resultados mostraram estabilidade ou melhora de desempenho no reteste na avaliação realizada com os monossílabos e 80% (n= 72) também demonstraram esse comportamento com os dissílabos.

**Figura 1:** Distribuição da diferença de resultado entre o teste e reteste no Teste de Palavras no Ruído com estímulo monossilábico (n=90)

**Legenda:** X: diferença entre os resultados no teste e reteste, expressa em dB;

Piora: diferença entre os resultados no teste e reteste menor que -1 dB;

Estabilidade: diferença entre os resultados no teste e reteste entre -1 dB e 1 dB;

Melhora: diferença entre os resultados no teste e reteste maior que 1 dB

**Figura 2:** Distribuição da diferença de resultado entre o teste e reteste no Teste de Palavras no Ruído com estímulo dissilábico (n=90)

**Legenda:** X: diferença entre os resultados no teste e reteste, expressa em dB;

Piora: diferença entre os resultados no teste e reteste menor que -1 dB;

Estabilidade: diferença entre os resultados no teste e reteste entre -1 dB e 1 dB;

Melhora: diferença entre os resultados no teste e reteste maior que 1 dB

Quanto à análise de confiabilidade no teste-reteste para o TPR (Tabela 2), os resultados demonstraram grau de confiabilidade bom para os monossílabos e moderado para os dissílabos, sendo estes considerados estatisticamente significantes.

**Tabela 2:** Análise da confiabilidade do Teste-Reteste do Teste de Palavras no Ruído

	ICC	Lim. Inferior	Lim. Superior	p-valor
Monossílabos	0,759	0,548	0,861	<0,001*
Dissílabos	0,631	0,441	0,757	<0,001*

Índice de Correlação Intraclasses (ICC)

* Valor estatisticamente significante no nível de 5% (p ≤ 0,05)

**Legenda:** Lim: limite

## DISCUSSÃO

Com o propósito de avaliar a confiabilidade do TPR, foi realizada a análise de desempenho dos sujeitos normo-ouvintes no teste-reteste. Tal análise é considerada um dos principais critérios de qualidade de um instrumento, sendo uma das estratégias mais utilizadas na área da saúde para verificação da confiabilidade^(^18^)^.

Ao analisar os resultados do TPR, foi possível constatar que, apesar da diferença significante observada entre o teste e reteste (Tabela 1), houve uma tendência de estabilidade nas medidas obtidas nos diferentes momentos da avaliação ou, então, de melhora dos resultados para ambos os estímulos, sendo que a concentração dos dados ocorreu entre -1 a +3 dB (Figuras 1 e 2).

A análise de confiabilidade no teste-reteste para o TPR (Tabela 2) foi realizada utilizando-se o Índice de Correlação Intraclasse, o qual é um dos principais testes estatísticos utilizado para estimar a estabilidade dos resultados de um instrumento, pois leva em consideração os erros de medida^(^26^)^. Os resultados demonstraram bom grau de concordância entre o desempenho dos sujeitos no teste e reteste para os monossílabos e moderado grau de concordância para os dissílabos.

Um estudo analisou a confiabilidade no teste e reteste de um instrumento que utiliza palavras monossilábicas para avaliação do reconhecimento de fala no ruído, intitulado *The Speech in Babble* (SiB) em adultos normo-ouvintes, por meio da aplicação do teste duas vezes na mesma orelha em um único momento de avaliação^(^27^)^ e não foi encontrada diferença estatisticamente significante entre os dois ensaios, tanto na orelha direita quanto na esquerda. Os pesquisadores afirmaram que a confiabilidade do teste-reteste foi “equivalente”.

A confiabilidade de um instrumento avaliada por meio do teste-reteste não é considerada uma propriedade fixa, de tal maneira que existem algumas fontes de erro que fogem do controle do avaliador. Nas avaliações de reteste, o ideal é que os sujeitos mantenham o desempenho da primeira avaliação^(^28^)^, porém as avaliações do reconhecimento de fala são extremamente sensíveis aos fatores intrínsecos de cada pessoa. Melhoras na segunda avaliação podem estar associadas à familiarização ao teste (estratégia de aplicação e estímulo), bem como à possibilidade de aprendizagem^(^20^)^. Já as pioras de desempenho no reteste podem estar associadas ao nível de atenção e concentração, falta de motivação pelo fato do teste já não ser mais uma novidade, estresse, entre outros fatores intrínsecos^(^20^)^.

Outra questão a ser considerada é o fato do TPR ser composto por palavras mono e dissilábicas, as quais são sensíveis às habilidades auditivas de cada sujeito. O estímulo de palavras representa pequenas unidades da fala, as quais apresentam certo contexto linguístico, são familiares, porém possuem poucas pistas acústicas e pouca redundância, o que faz com que os sujeitos necessitem escutar a maioria de seus elementos para reconhecê-las^(^29^)^. Dessa forma, palavras são mais suscetíveis a erros em função de fatores intrínsecos, como questões atencionais e de concentração, pois a “perda” da informação acústica de um único fonema já dificulta o reconhecimento da palavra.

Fatores metodológicos também podem influenciar os resultados do teste-reteste, tais como o intervalo entre a primeira e segunda avaliação e o tamanho amostral. Buscando diminuir a influência destes fatores, neste estudo levou-se em consideração as principais recomendações metodológicas para a pesquisa da confiabilidade do instrumento^(^18,20,28^)^.

Em relação ao intervalo entre teste e reteste, sabe-se que o período entre a repetição das testagens deve ser longo o suficiente para impedir o efeito da memória, mas curto o suficiente para garantir que não ocorram alterações clínicas, influenciando sua interpretação^(^18,20^)^. Desse modo, escolheu-se um intervalo de tempo de 14 a 28 dias, considerado adequado para esse fim.

Quanto ao tamanho amostral, este estudo contou com uma amostra de 45 sujeitos avaliados, aproximando-se da recomendação de pesquisadores que sugerem amostras superiores a 50 participantes para avaliação da confiabilidade do teste-reteste^(^28^)^ e superando a recomendação mínima de 20 sujeitos para análise do Coeficiente de Correlação Intraclasse^(^30^)^.

Vale ressaltar que apesar da variabilidade observada na segunda avaliação, a análise de correlação revelou bom grau de concordância entre o teste e reteste para o TPR-M, e moderado grau de concordância para o TPR-D. Isso indica confiabilidade aceitável dos resultados obtidos com o TPR, considerando a variabilidade inerente dos instrumentos de avaliação do reconhecimento de fala e também o objetivo principal de aplicação do TPR na rotina clínica, que é a de desempenhar a função de um instrumento capaz de identificar a dificuldade de um sujeito para reconhecer a fala no ruído e, a partir de seus resultados, verificar a necessidade de avaliações complementares.

Entretanto, se o examinador tiver como objetivo o seu uso em uma situação de comparação pré e pós-treinamento auditivo ou adaptação de amplificação sonora deve levar em consideração que um sujeito, ao ser reavaliado com um teste com tais características, pode apresentar variabilidade de desempenho na segunda avaliação. Portanto, seus resultados devem ser analisados com cautela.

Sobre as limitações do estudo, é possível elencar a dificuldade de controlar as condições intrínsecas de cada sujeito, tais como estado emocional, atenção e concentração, as quais podem interferir nos resultados de um teste comportamental quando é necessário que esse seja aplicado em momentos distintos de avaliação.

## CONCLUSÃO

O Teste de Palavras no Ruído demonstrou satisfatório grau de confiabilidade no teste-reteste em adultos normo-ouvintes, tanto para o estímulo monossilábico quanto para o estímulo dissilábico.
